# Ethnic Minorities with Diabetes Differ in Depressive and Anxiety Symptoms and Diabetes-Distress

**DOI:** 10.1155/2017/1204237

**Published:** 2017-03-08

**Authors:** Charlotte B. Schmidt, Bert Jan Potter van Loon, Bart Torensma, Frank J. Snoek, Adriaan Honig

**Affiliations:** ^1^Department of Psychiatry, Onze Lieve Vrouwe Gasthuis (OLVG), Amsterdam, Netherlands; ^2^Amsterdam Public Health Research Institute, Amsterdam, Netherlands; ^3^Department of Internal Medicine, Onze Lieve Vrouwe Gasthuis (OLVG), Amsterdam, Netherlands; ^4^Department of Epidemiology and Biostatistics, Onze Lieve Vrouwe Gasthuis OLVG, Amsterdam, Netherlands; ^5^Department of Medical Psychology, Academic Medical Centre (AMC), Amsterdam, Netherlands; ^6^Department of Medical Psychology, VU University Medical Centre (VUMC), Amsterdam, Netherlands; ^7^Department of Psychiatry, VU Medical Centre (VUmc), Amsterdam, Netherlands

## Abstract

*Objective*. To determine the association between ethnicity, diabetes-distress, and depressive and anxiety symptoms in adult outpatients with diabetes.* Research Design and Methods*. Diabetes-distress (Problem Areas in Diabetes Scale, PAID5), depressive and anxiety symptoms (Extended Kessler-10, EK10), and quality of life (Short-Form 12, SF12) were assessed in an ethnic diverse diabetes outpatient population of a teaching hospital in Amsterdam. Descent of one's parents and self-classified ethnicity were obtained to define ethnicity. HbA1c, clinical data, and socioeconomic status were derived from the medical charts. Based on established cut-offs for PAID5- and EK10-scores, emotional distress was dichotomized for the purpose of logistic regression analyses.* Results*. Of 1007 consecutive patients approached, 575 participated. Forty-nine percent were of non-Dutch ethnicity and 24.7% had type 1 diabetes. Diabetes-distress was reported by 12.5% of the native Dutch patients and by 22.0%, 34.5%, and 42.6% of the Surinamese, Turkish, and Moroccan patients, respectively. Prevalence of depressive symptoms was 9.4% in native Dutch patients and 20.4%, 34.5%, and 27.3% in the other groups mentioned. Diabetes-distress and Moroccan origin were significantly associated (OR = 3.60, *p* < .01) as well as depressive symptoms and Turkish origin (OR = 4.23, *p* = .04).* Conclusions*. Different ethnic minorities with diabetes vary in their vulnerability for emotional distress, warranting clinical attention. Future research should elucidate explanatory factors and opportunities for tailored interventions.

## 1. Introduction

Emotional distress is common in persons with diabetes [1]. It is associated with poorer glycaemic control, reduced quality of life, and higher mortality rates [2–4]. Emotional distress can either be generic, defined as depressive and/or anxiety symptoms not related to a specific cause, or diabetes-specific, that is, directly related to the experience of living with diabetes mellitus, such as fear of complications or worrying about the disease and its direct consequences for daily life [1]. Both generic distress (depressive and anxiety symptoms) and diabetes-distress are highly prevalent in persons with diabetes [5], in particular in secondary and tertiary care [6]. Overall, persons with diabetes report depressive symptoms twice as often as the general population [7] and anxiety symptoms are present in 27%–40% of persons with diabetes [8].

Predictors of depressive symptoms and diabetes-distress were female sex, life events, and concomitant diseases in a mixed sample of type 1 and type 2 outpatients with diabetes across 8 countries [9]. Poor glycaemic control was a predictor of diabetes-distress, but not of depressive symptoms [9]. These findings are in line with earlier findings in the US [10]. In recent years, given the increasing ethnic diversity among diabetes patients in the Netherlands and other European countries, ethnicity has attracted a growing attention, more so, given the evidence that health outcomes overall are poor in ethnic minorities in Western societies [11].

In the past decades, predominately immigrants with little to no formal education from Morocco and Turkey came to the Netherlands as guest workers, followed by their family members [12]. Also inhabitants from its former colony Suriname have migrated to the Netherlands, most of them from South Asian, African, or mixed descent. Diabetes is more prevalent among non-Western minorities compared to the indigenous population in North-West Europe [13]. In the Netherlands, in particular Turkish and Moroccan migrants are more prone to developing diabetes, which cannot sufficiently be explained by socioeconomic status or BMI [14]. Ethnic minorities with diabetes in the US and Asians in the UK are more prone to develop end-stage renal disease in comparison to white diabetes patients, while blacks and Hispanics in the US have an increased risk of retinopathy [15, 16].

Similar to diabetes and its complications, the prevalence of emotional distress differs between ethnic subgroups. In persons without diabetes, Turkish or Moroccan minority status in both general Western population [17] and Dutch hospital settings is associated with increased rates of depression [18]. In persons with diabetes in UK secondary care, Ali et al. (2009) found that South-Asians report less depressive symptoms than white persons with diabetes [19]. A similar conclusion was drawn by Fisher and colleagues in the US: Asians and African-Americans with diabetes less often suffer from depressive disorder than non-Hispanic Caucasians with diabetes [20]. In contrast, in an ethnic diverse Dutch community sample Pouwer et al. (2013) found that one-third of the persons with diabetes experienced elevated depressive and anxiety symptoms, unrelated to ethnicity [21]. These findings were however based on a relative small cohort (*n* = 140, divided into four groups), warranting replication in a larger sample of ethnic diversity. A larger cohort of diabetes patients in the Netherlands (*n* = 864) showed a higher prevalence of depressive symptoms in a combined group of migrants from Turkish, Moroccan, Surinamese, and Indonesian descent [22]. Overall, the prevalence of depressive symptoms seems to differ in ethnic minorities.

In line with findings regarding depression, the overall prevalence of anxiety in a Dutch general hospital setting was 51.7% for ethnic minorities and 36.6% in native Dutch [18]. In a large population based sample in the US, anxiety was related to ethnicity in patients with diabetes [23]. Hispanics and non-Hispanic blacks reported more anxiety symptoms than other ethnic backgrounds.

Similar differences are found in the prevalence of diabetes-distress in ethnic minorities. Both in the US and in the Netherlands, the combined group of ethnic minorities with diabetes experienced more diabetes-distress compared to white persons with diabetes [6, 24]. Similar to depressive and anxiety symptoms this association might differ across ethnic subgroups. Findings from Peyrot et al. (2014) support this hypothesis. In an ethnic diverse sample of diabetes patients in the US, African-Americans reported the lowest depressive symptoms, while Hispanics reported the highest diabetes-distress [24].

We do not know whether ethnic differences similar to those in the US exist in patients with diabetes in Europe, nor do we know which factors could explain possible disparities with relation to emotional distress. Elucidation of these associations could help in developing tailored interventions. Further exploration is required, considering established risk factors for emotional distress. We therefore set out to study the association between ethnicity and emotional distress in an ethnic diverse diabetes patient population.

Our aim was, first, to test the association between ethnicity and depressive and anxiety symptoms and diabetes-distress, with the expectation to find confirmation for the association between ethnic minorities and elevated depressive and anxiety symptoms and diabetes-distress [6, 17, 18].

Our second aim was to investigate whether these associations differ between different ethnic subgroups. Based on research to date, we hypothesized that in particular Turkish and Moroccan persons would report relatively high levels of depressive and anxiety symptoms [18]. As to the between-ethnic differences in diabetes-distress we had no hypothesis.

## 2. Participants and Methods

This observational cohort study was conducted in the diabetes outpatient clinic of the Onze Lieve Vrouwe Gasthuis (OLVG), Amsterdam, the Netherlands. The clinic serves a population of 1500 patients, with approximately 30% having a non-Dutch ethnic background. Consecutive persons with diagnosed diabetes were invited by physician assistants to participate in the study when visiting the diabetes outpatient clinic. Participation was voluntary: no incentive or compensation was provided. All participants provided written informed consent. Ethical approval of the study was obtained from the Institutional Review Board of OLVG.

### 2.1. Participants

Consecutive adult (≥18 years) persons of the diabetes clinic were screened. People with all types of diabetes were eligible, except women diagnosed with gestational diabetes. Persons with language difficulties were excluded, determined as being unable to give informed consent.

### 2.2. Questionnaires

Prior to consultation, physician assistants requested the eligible persons to complete a paper questionnaire booklet in Dutch, Turkish, Arabic, or English. The following variables were measured.

#### 2.2.1. Depressive and Anxiety Symptoms

Depressive and anxiety symptoms were measured by means of the Dutch version of the EK10 (Extended Kessler-10) [25], an extended version of the Kessler-10; Cronbach's *α* for the EK10 is 0.94 [25]. This screening tool, used to determine probable depression or anxiety disorders, has both high sensitivity (0.90) and specificity (0.75) [25]. We chose this questionnaire since it is well studied in ethnic minorities in the Netherlands. The EK10 contains 10 items about mood on a 5-point Likert scale (scored from 0 to 4, in which 0 stands for “never” and 4 stands for “all the time”) and 5 items about anxiety with a “yes” (scored as 1), or “no” (scored as 0), scoring (applicable or nonapplicable). All questions pertain to depressive and anxiety symptoms during the past month. A cut-off score of 20, out of a maximum of 40, was used to indicate depressive symptoms. To indicate anxiety symptoms, a cut-off score of 1 was used.

#### 2.2.2. Diabetes-Distress

Diabetes-distress, defined as emotional distress directly associated with diabetes mellitus and its treatment, was measured by means of the Dutch version of the PAID5 (Problem Areas in Diabetes Short Form) [26]. Cronbach's *α* of the PAID5 ranges from 0.84 to 0.88, and the Dutch and US version are psychometrically equivalent, which allows cross-cultural comparisons [26, 27]. It has both high sensitivity (0.94) and specificity (0.89). The PAID5 consists of 5 items (items 3, 6, 12, 16, and 19) derived from the PAID20. These 5 items pertain to worrying about the future and risk of complications, feeling scared or feeling depressed when thinking of diabetes, feeling diabetes is taking up too much of mental and physical energy, and coping with complications. The items are measured on a 5-point Likert scale, scoring from 0 to 4 in which 0 stands for “not a problem” and 4 stands for “a serious problem.” A higher score indicates more diabetes-distress. A cut-off score of 8, out of a maximum of 20, was used to indicate elevated diabetes-distress.

#### 2.2.3. Quality of Life

Quality of life was measured by means of the Dutch version of the SF12 (Health Survey Short Version) [28]; Cronbach's *α* of the SF12 ranges from 0.91 to 0.94 [29]. It has a sensitivity of 0.88 and a specificity of 0.89 and is well examined in patients with diabetes [30, 31]. The SF12 contains 12 items derived from the SF-36 that asks about the perceived mental and physical health in the past 4 weeks. It measures different domains including physical functioning, bodily pain, general health, vitality, social functioning, role functioning emotional, and mental health. Results can be summated into two composite scores: physical and mental health [29].

#### 2.2.4. Ethnicity

Data on ethnicity were collected by a 9-item questionnaire. One's ethnicity was determined based on the patient's reported birth country of one's parents. If both parents were born in a different country, we used the descent of the mother. Country of origin is considered a useful method for defining ethnicity in the Netherlands, as it highly correlates with self-classified ethnicity [32]. In addition, self-classified ethnicity was obtained by means of a single item question: “To which ethnic group do you belong?”. This is in line with Bhopal's (2004) glossary on ethnicity and race, which states that ethnicity should preferably be classified based on different classification methods [33]. Ethnicity was classified into five groups for additional analyses: native Dutch, Moroccan, Turkish, Surinamese, and Other, defined as all other ethnic backgrounds. For nonrespondents, ethnicity was estimated based on last name, by a medical specialist of the clinic.

#### 2.2.5. Somatic and Socioeconomic Status

Biological and other parameters (HbA1c, comorbidity, complications, BMI, age, type of diabetes, and gender) were obtained from the medical charts. Socioeconomic status was estimated based on postal area code in a dichotomous way (0 = nonlow socioeconomic status, 1 = low socioeconomic status). These estimations were based on the postal area codes listed in a national registry to income, population density, mean % of unemployment (excluding students), and mean educational level [34].

### 2.3. Statistical Analyses

Predictor-outcome ratio was calculated with the following formula: *N* = 10*k*/*p*, in which* k* stands for the number of predictors and* p *stands for the proportion of high (≥8) PAID-scores in the population of persons with diabetes [35]. In this case* p* is 0.15 [5] and* k* is 8: ethnicity, depressive and anxiety symptoms, quality of life, socioeconomic status, HbA1c, gender, age, and BMI. This results in a sample size of around 533 persons to register significant associations.

For analyses we used descriptive statistics and inferential statistics. Missing data were imputed by multiple imputation when up to 3 questions per questionnaire were missing; otherwise the patient was excluded from additional analyses [36]. All data were first tested for normality by a Kolmogorov-Smirnov test, a Q-Q plot, and Levene's test. Categorical variables were expressed as* n* (%). Continuous normally distributed variables were expressed by their mean and standard deviation, for skewed distributions by their median and interquartile range. Normally distributed continuous data were tested with the independent samples Student's *t*-test and, in case of skewed data, with the independent samples Mann–Whitney *U* test. Mean differences in depression, anxiety and diabetes-distress were tested with one-way ANOVA, grouped by ethnicity. We used post hoc tests (Bonferroni) to tell which groups differed from the rest. Predictors of depression, anxiety, and diabetes-distress were evaluated using univariate and multivariable logistic regression analysis. Based on established cut-off scores indicating depression or not, anxiety or not, and diabetes-distress or not [25, 27] outcome variables were dichotomized for the purpose of logistic regression analyses. We checked for multicollinearity to make sure all variables measured different outcomes and to ascertain the variables were not strongly correlated. If the Variance Inflation Factors were <1.5, no multicollinearity was detected and all variables could be used as independent variables [37]. Possible moderating and mediation effects were calculated according to Baron and Kenny (1986) [38], followed by a Sobel test [39].

All independent variables counting more than ten events and showing* p* values < .10 were eligible for multivariable analysis, which was achieved through the enter method. The optimal prediction model was evaluated with −2 log likelihood. Significance level for baseline variables and multivariable regression analysis was set at* p* value < .05. Statistical analysis was performed using SPSS Statistical software (version 21.0, SPSS Inc., Chicago, IL).

## 3. Results

The study population consisted of 1007 consecutively asked persons (70% of total population served), of which 203 were excluded because of language difficulties and 229 refused to participate (8 persons had vision problems; other reasons for refusing were not specified), leaving 575 participants in the study. Since most persons with language difficulties were illiterate, we decided to solely hand out questionnaires in Dutch. If persons were unable to fill these in, they were helped by family members or the interviewer. No significant differences were observed between refusers and included persons in HbA1c-levels and gender (Table 1). Both refusers and persons with language difficulties had lower socioeconomic status and higher BMI than respondents (*p* < .05). Refusers were older than respondents (*p* < .05), while persons with language difficulties had higher HbA1c than respondents (*p* < .05). Since ethnicity was not reported in the medical records, these data were not available for nonrespondents. Therefore ethnicity of nonparticipants was estimated based on the person's last name, by a medical specialist of the clinic. Based on these estimations, approximately 60% of the total group of nonrespondents (refusers and language difficulties) were of combined ethnic minority. Of these nonnative Dutch nonrespondents approximately 50% were Moroccan (compared to 26% of the respondents), 24% were Turkish (12% of the respondents), 3% were Surinamese (23% of the respondents), and 23% were of other descent (34% of respondents). Persons that declined to participate due to language difficulties and the combined group of participants from ethnic minority had similar HbA1c-levels, BMI, socioeconomic status, and gender, data not shown.

Table 2 shows the demographic characteristics of the 575 included persons. Mean age was 58.5 ± 14 years, 55.3% were men, and 55.1% had 1 or more comorbid diseases. The ethnic subgroups consisted of 49.3% native Dutch and 48% combined ethnic minority. Ethnicity was not reported by 2.7% of the included group. Mean HbA1c-level was 7.8% (62 mmol/mol) and mean BMI 30.1 ± 6.5 kg/m^2^. Turkish persons had the highest HbA1c-levels compared to other ethnic backgrounds (*p* < .01) (Table 2). Both Turkish and Moroccan persons had lower socioeconomic status in comparison to other ethnic backgrounds (*p* < .01) (Table 2).

### 3.1. Ethnicity and EK10- and PAID5-Scores

First, the prevalence of diabetes-distress (PAID5 ≥ 8) was 12.5% among native Dutch persons and 22.0%, 34.5%, and 42.6% among Surinamese, Turkish, and Moroccan persons, respectively. Second, the prevalence of depressive symptoms (EK10 depression ≥ 20) was 9.4% among native Dutch people and 20.4%, 27.3%, and 34.5% among Surinamese, Moroccan, and Turkish people, respectively. Third, the prevalence of anxiety symptoms (EK10 anxiety ≥ 1) was 33.8% among native Dutch persons and 53.1%, 54.0%, and 60.3% in Turkish, Surinamese, and Moroccan persons, respectively (Table 2).

One-way ANOVA showed higher depressive and anxiety symptoms and diabetes-distress for ethnic minorities compared to native Dutch (*p* < .001), as reported in Table 3. Bonferroni post hoc analyses showed that both Moroccan and Turkish persons reported more depressive and anxiety symptoms and diabetes-distress compared to native Dutch (*p* < .01), while other ethnic backgrounds did not (*p* > .05). First, the mean depressive symptoms score was 8.8 (7.4) for native Dutch persons, compared to 15.5 (9.8) and 16.1 (10.4) for Moroccan and Turkish persons, respectively. Second, the mean anxiety symptoms score was 0.5 (0.9) for native Dutch persons, in comparison to 1.2 (1.4) and 1.4 (1.5) for Turkish and Moroccan persons, respectively. Third, mean diabetes-distress was 3.2 (3.9) for native Dutch persons, compared to 6.1 (5.0) and 7.1 (5.3) for Turkish and Moroccan persons, respectively.

Moroccan and Turkish ethnicity was thus related to both diabetes-distress and depressive and anxiety symptoms. We sought to determine whether these associations were explained by putative confounders, by using multivariable logistic regression analyses.

### 3.2. Diabetes-Distress

Possible predictors (*p* < .10) for diabetes-distress were Moroccan and Turkish ethnicity, socioeconomic status, age, physical quality of life, EK10 depression, EK10 anxiety, and HbA1c.

Multivariable logistic regression analysis, reported in Table 4, demonstrated a significant association between PAID5 and Moroccan origin (OR = 3.60; 95% CI = 1.37 to 9.45; *p* < .01). This indicates that the odds of diabetes-distress are 3.60 for Moroccans compared to native Dutch. Other ethnic backgrounds and PAID5 were not associated in the multivariable analysis.

### 3.3. Depressive and Anxiety Symptoms

Possible predictors (*p* < .10) for depressive symptoms were Moroccan, Turkish, and Surinamese ethnicity, socioeconomic status, age, physical quality of life, PAID5, HbA1c, and EK10 anxiety.

**Figure 1:**
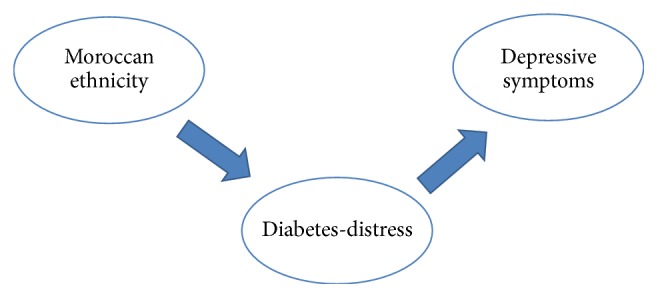
The mediating effect of diabetes-distress between Moroccan ethnicity and depressive symptoms.

Turkish origin and EK10 depression were significantly associated in multivariable logistic regression analysis reported in Table 5 indicating that the odds of depressive symptoms are 4.23 for Turkish persons compared to native Dutch (OR = 4.23; 95% CI = 1.05 to 17.04; *p* = .04). In unadjusted analyses, Moroccan origin was also associated with EK10 depression (OR = 3.54, *p* < .01). However, when PAID5 was included in the model, Moroccan origin was no longer significantly associated with EK10 depression (OR = 1.69, *p* = .21). According to a Sobel test, PAID5 significantly mediated the association between Moroccan origin and EK10 depression (*p* < .01), suggesting an important role for diabetes-distress in this ethnic group (Figure 1). No association was found between other ethnic backgrounds and EK10 depression.

**Figure 2:**
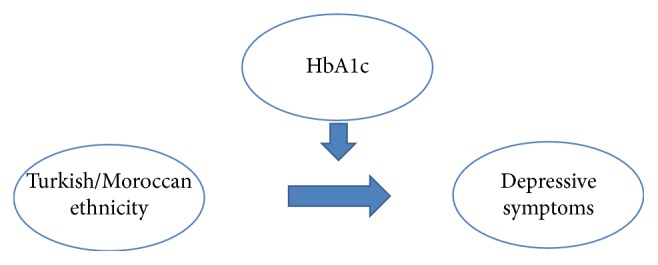
The interaction effect between Moroccan and Turkish ethnicity, HbA1c, and depressive symptoms.

In addition, there was an interaction effect between both Turkish and Moroccan ethnicity, HbA1c and EK10 depression (Figure 2). Turkish and Moroccan minorities with high HbA1c-levels reported depressive symptoms more often than Turkish and Moroccan minorities with low HbA1c-levels.

With respect to anxiety symptoms, possible predictors (*p* < .10) were Moroccan, Turkish, and Surinamese ethnicity, socioeconomic status, physical quality of life, PAID5, HbA1c, EK10 depression, number of complications, and gender.

Multivariable logistic regression analysis reported in Table 6 demonstrated no significant association between EK10 anxiety and ethnicity, in contrast to socioeconomic status and HbA1c.

## 4. Discussion

The objective of this study was to investigate whether ethnicity was associated with diabetes-distress and depressive and anxiety symptoms, taking different ethnic backgrounds into consideration. We hypothesized that all ethnic minorities would report higher levels of emotional distress in comparison to native Dutch persons and that in particular Turkish and Moroccan persons would report high levels of depression and anxiety [18]. As to the between-ethnic differences in diabetes-distress we had no hypothesis. Our findings add to the literature on the differential associations between ethnic subgroups and types of emotional distress in diabetes.

In accordance with our hypotheses, diabetes-distress and depressive and anxiety symptoms were more prevalent among ethnic minorities compared to native Dutch persons. Turkish persons reported the most depressive symptoms, while Moroccans reported the most diabetes-distress.

Our prevalence rates are in line with findings from a systematic review from Roy and Lloyd (2012), which states that persons with diabetes are 2-3 times more likely to develop depression compared to persons without diabetes [7]. The ethnic differences are in concordance with previous findings in the general population in the Netherlands [40]. Also the total prevalence of anxiety in our cohort is in line with previous findings in patients with diabetes [8].

Ethnic minorities had lower socioeconomic status and higher HbA1c than indigenous persons in our sample. Previous research indicates that these two elements are associated with diabetes-distress and depressive and anxiety symptoms [3, 41, 42]. These findings were reproduced in our sample: according to unadjusted analyses, HbA1c and socioeconomic status were associated with both depressive and anxiety symptoms and diabetes-distress. However, adjusted analyses revealed that ethnicity explained the associations between socioeconomic status, HbA1c and depressive symptoms, and diabetes-distress. In contrast, ethnicity did not account for the associations between socioeconomic status, HbA1c, and anxiety symptoms.

While all ethnic minorities reported more emotional distress than native Dutch persons, the associations differed between ethnic subgroups as postulated. In summary, Surinamese reported the least depressive symptoms and diabetes-distress compared to other ethnic minorities. Moroccans reported more diabetes-distress than other ethnic backgrounds, and Turkish persons reported more depressive symptoms than other ethnic minorities, corroborating previous studies in general Dutch and Turkish population in both the Netherlands and Turkey [40, 43].

These findings are partly in line with our hypotheses: it was expected that in particular Moroccans and Turkish persons would report high levels of depressive and anxiety symptoms. We did, however, not expect discrepancies between Moroccans and Turkish persons in the prevalence of depressive symptoms and diabetes-distress. While Moroccans more often reported depressive symptoms than other ethnic backgrounds, this was explained by the fact that they experienced more diabetes-distress (Figure 1). This did not apply to Turkish persons, indicating that somehow Moroccans seem more affected by diabetes-distress.

Furthermore, Turkish and Moroccan minorities with high HbA1c-levels reported depressive symptoms more often than Turkish/Moroccan minorities with low HbA1c-levels (Figure 2). This suggests an important role for HbA1c in Moroccan and Turkish persons.

Whereas our findings on depressive symptoms could reflect the prevalence of these symptoms in general population [40], this does not unravel the differential associations between types of emotional distress and subgroups of ethnic minorities. It remains unclear which underlying mechanisms can explain the differential ethnicity-specific associations with emotional distress. We can assume that ethnic differences in emotional distress might be affected by religion and religious coping strategies. Moroccan and Turkish persons are often Muslim, in contrast to Surinamese persons. Muslims consider religious coping behaviour a more appropriate response to emotional distress than seeking social support or professional help [44]. Expressing emotional distress may be seen as a sign of weakness and surrounded with stigma, leading to more use of “private” coping strategies [45]. These differences in religion and coping could partly explain ethnic differences in emotional distress. However, this does not help explain the observed differential associations between Turkish and Moroccan minority and depressive symptoms and diabetes-distress.

Differences in symptom presentation may be affected by culture and could explain these differential associations. Al-Krenawi (2005) described that persons in Arab countries (including Morocco) often present their psychological problems in terms of physical symptoms [46]. This could offer a possible explanation for the association between Moroccans and diabetes-distress, since items of the PAID5 are specific to diabetes, while items of the EK10 (general distress) are generic. We therefore speculate that symptom presentation could play a role in the association between Moroccan ethnicity and diabetes-distress. Moreover, previous findings suggest that acculturation (integration, assimilation, separation, and marginalization) is related to depressive symptoms in Turkish minorities in the Netherlands [47]. Unfortunately, we do not have data on acculturation to verify this possible explanation, nor do we know whether acculturation is related to depressive symptoms in Moroccan minorities. Further research is warranted to elucidate explanatory factors of the differential associations between Turkish and Moroccan minority and depressive symptoms and diabetes-distress.

*Limitations and Strengths*. Some limitations of our study need to be mentioned. First, the cross-sectional design of the study makes it impossible to draw causal conclusions. The observed associations might not be causal, due to either unknown confounders or reverse causation. Although our multivariable logistic analyses were adjusted for possible confounders including, for example, gender, comorbidity, and socioeconomic status, we cannot rule out the possibility of confounders that were not included.

Second, it is plausible that selection bias has occurred since 42% of the approached group did not participate. One group refused to participate (22%); a second group had language problems (20%). Although we tried to overcome the language barrier by handing out questionnaires in Arabic, Turkish, and English, we had to exclude people who were illiterate. Both refusers and persons with language difficulties differed from the respondents (Table 1), indicating selection bias. This limits the external validity of our study findings that therefore cannot be generalized to the total population of patients with diabetes in secondary care.

For nonrespondents, we had to estimate ethnicity based on the person's last name. This is not the most precise measure, but accurate enough to allow for identification on a group level. The ones that declined to participate because of language problems were most likely from an ethnic minority. This hypothesis is supported by the fact that the combined group of respondents from ethnic minorities had similar HbA1c-levels, BMI, socioeconomic status, and gender compared to nonrespondents with language difficulties, data not shown.

Language problems are associated with depressive symptoms and no-show is known to be high among persons with a current depressive episode [48, 49]. Nonrespondents in our sample had lower socioeconomic status and higher HbA1c than respondents, while these factors are associated with depression and diabetes-distress [3, 50]. This suggests that persons with depression and diabetes-distress might be underrepresented in this study, that is, depressive symptoms and diabetes-distress in secondary diabetes care could be more prevalent than reported.

Both the response rate (58%) and the differences between respondents and nonrespondents were in line with findings from Van Bastelaar et al. (2010) in a similar patient sample [51]. This illustrates that the issue of selective response is probably inherent to research in this patient population. However, the results were not affected by nonrepresentativeness of these factors since we controlled for socioeconomic status, BMI, HbA1c, age, and other putative confounders in all analyses.

A third limitation of our study is the determination of socioeconomic status based on area level postal codes. However, previous research suggests that it is an acceptable technique [52]. As a result of the large number of excluded persons, our conclusions are based on small sample sized ethnic subgroups. Nevertheless, our conclusions are in line with a previous US study in larger groups [24].

The ethnic differences in emotional distress underpin the importance of developing a culturally sensitive framework for understanding depressive symptoms and diabetes-distress in patients with diabetes. Our results suggest that there are differential mechanisms other than, for example, socioeconomic status underlying depressive symptoms or diabetes-distress in Turkish and Moroccan immigrants, which cannot be generalized from one ethnic group to another [24]. More research is warranted to further elucidate the differences in risk/protective factors for emotional distress across the different groups and the implications for clinical practice.

In conclusion, ethnic minorities with diabetes differ in their vulnerability for emotional distress, warranting clinical attention. Our findings underscore the importance of adequate management of high emotional distress in ethnic patients taking the different distress profiles into account.

## Figures and Tables

**Table 1:** Demographic characteristics of respondents (*n* = 575) and nonrespondents (*n* = 432) divided by reason of nonresponding.

Characteristic	Respondents (*n* = 575)	Combined nonrespondents (*n* = 432)	Refusers (*n* = 203)	Language difficulties (*n* = 229)
	*n *(%) or mean ± SD	*n *(%) or mean ± SD	*n *(%) or mean ± SD	*n *(%) or mean ± SD
Gender				
Men	315 (55.3%)	237 (54.9%)	106 (52.2%)	131 (57.2%)
Women	255 (44.7%)	195 (45.1%)	97 (47.8%)	98 (42.8%)
Mean age (SD)	58.5 (14.0)	61.9 (13.0)^a^	63.7 (13.3)^a^	60.4 (12.6)
HbA1c % (mmol/mol)	7.8% (62)	8.1% (65)^a^	7.9% (63)	8.3% (67)^a^
BMI	30.1 (6.5)	31.4 (6.3)^a^	31.1 (6.2)^a^	31.7 (6.5)^a^
Socioeconomic status				
Nonlow	360 (62.5%)	199 (48.5%)^a^	108 (54.8%)^a^	91 (42.7%)^a^
Low	205 (35.6%)	211 (51.5%)	89 (45.2%)	122 (57.3%)

^a^ *p* < .05, compared to respondents.

**Table 2:** Demographic characteristics and prevalence rates of high EK10- and PAID5-scores of respondents divided by ethnicity (*n* = 575).

Characteristic	Overall (*n* = 575)	Native Dutch (*n* = 284)	Moroccan (*n* = 73)	Turkish (*n* = 32)	Surinamese (*n* = 63)	Other^c^ (*n* = 108)	Missing^a^ (*n* = 10)
	*n *(%) or mean (SD)	*n *(%) or mean (SD)	*n *(%) or mean (SD)	*n *(%) or mean (SD)	*n *(%) or mean (SD)	*n *(%) or mean (SD)	
Gender							
Men	315 (55.3%)	147 (51.8%)	43 (58.9%)	17 (53.1%)	34 (54.0%)	65 (60.2%)	
Women	255 (44.7%)	137 (48.2%)	30 (41.1%)	15 (46.9%)	29 (46.0%)	43 (39.8%)	
Mean age (SD)	58.5 (14.0)	60.4 (15.2)	52.2 (13.9)^d^	52.2 (15.5)^d^	59.1 (11.8)	59.6 (12.9)	
HbA1c % (mmol/mol)	7.8% (62)	7.7% (61)	8.0% (64)	8.6% (70)^d^	8.3% (67)^d^	7.6% (60)	
BMI	30.1 (6.5)	29.6 (6.7)	30.3 (7.2)	34.7 (5.9)^e^	30.4 (5.2)	29.8 (6.2)	
Socioeconomic status							
Nonlow	360 (62.5%)	205 (72.2%)	24 (32.9%)	12 (37.5%)	41 (65.1%)	74 (68.5%)	
Low	205 (35.6%)	79 (27.8%)	49 (67.1%)^d^	19 (59.4%)^d^	22 (34.9%)	34 (31.5%)	
Type 1 DM	140 (24.5%)	93 (32.7%)	21 (28.8%)	4 (12.5%)	6 (9.5%)	15 (14.2%)	
Type 2 DM	422 (74.8%)	189 (66.3%)	52 (71.2%)	28 (87.5%)	57 (90.5%)^d^	89 (84.0%)^d^	
LADA^b^	4 (0.7%)	2 (0.7%)	—	—	—	2 (1.9%)	
Comorbid diseases							
None	251 (43.3%)	126 (44.5%)	37 (52.9%)	17 (53.1%)	19 (30.6%)	42 (40.0%)	
1 or more	324 (55.1%)	157 (55.5%)	33 (47.1%)	15 (46.9%)	43 (69.4%)	63 (60.0%)	
Complications							
None	208 (36.2%)	105 (37.1%)	24 (33.3%)	16 (50.0%)	19 (30.6%)	37 (34.6%)	
1	218 (37.9%)	100 (35.3%)	34 (47.2%)	8 (25.0%)	24 (38.7%)	45 (42.1%)	
2 or more	149 (25.9%)	78 (27.6%)	14 (19.4%)	8 (25.0%)	19 (30.6%)	25 (23.3%)	
EK10 depression ≥ 20 (%)	13.5%	9.4%	27.3%	34.5%	20.4%	6.3%	
EK10 anxiety ≥ 1 (%)	37.8%	32.1%	57.6%	51.6%	50.8%	29.1%	
PAID5 ≥ 8 (%)	19.3%	12.5%	42.6%	34.5%	22.0%	15.4%	

^a^Did not report ethnicity in questionnaire.

^b^Latent autoimmune diabetes in adults.

^c^All other reported ethnic backgrounds.

^d^ *p* < .05 compared to native Dutch.

^e^ *p* < .05 compared to all other ethnic backgrounds.

**Table 3:** Mean EK10- and PAID5-scores, standard deviations, *F* tests, and effect sizes for native Dutch compared to other ethnic groups.

Measures	Study groups									
	(1) Native Dutch	(2) Moroccan	(3) Turkish	(4) Surinamese	(5) Other^c^	Effect size Cohen's *d*^a^				
	*n* = 281	*n* = 70	*n* = 31	*n* = 62	*n* = 108					
	M (SD)	M (SD)	M (SD)	M (SD)	M (SD)	*F* (4,547)	*d*2	*d*3	*d*4	*d*5
EK10-depression	8.8 (7.4)	15.5 (9.8)	16.1 (10.4)	11.8 (8.7)	9.2 (7.8)	14.4^b^	0.77	0.81	0.37	0.05
EK10-anxiety	0.5 (0.9)	1.4 (1.5)	1.2 (1.4)	0.9 (1.2)	0.5 (1.0)	11.9^b^	0.73	0.59	0.38	0
PAID5	3.2 (3.9)	7.1 (5.3)	6.1 (5.0)	4.8 (4.5)	3.9 (4.5)	13.5^b^	0.84	0.65	0.38	0.17

^a^Compared to native Dutch as the reference group.

^b^ *p* < .001.

^c^All other reported ethnic backgrounds.

**Table 4:** Unadjusted and adjusted logistic regression analyses of variables associated with diabetes-distress (PAID5).

Predictor	Unadjusted OR	95% C.I.		Adjusted OR	95% CI	
		Lower	Upper		Lower	Upper
Ethnicity						
Moroccan	*5.10* ^b^	2.80	9.27	*3.60* ^b^	1.37	9.45
Turkish	*3.70* ^b^	1.59	8.62	1.76	0.49	6.29
Surinamese	1.99	0.97	4.05	1.47	0.52	4.15
Other	1.28	0.67	2.43	1.33	0.57	3.09
Socioeconomic status	*1.62* ^b^	1.05	2.50	0.68	0.32	1.36
Age	*.97* ^b^	0.96	0.99	*0.97* ^b^	0.95	0.99
PCS (SF12)	*2.38* ^b^	1.44	3.94	*0.98* ^a^	0.96	1.00
MCS (SF12)	1.24	0.76	2.01			
EK10-depression	*10.43* ^b^	5.91	18.40	*6.79* ^b^	3.04	15.20
EK10-anxiety	*6.56* ^b^	4.05	10.62	*2.54* ^b^	1.28	5.03
HbA1c	*1.01* ^a^	1.00	1.03	1.00	0.98	1.02
BMI	1.02	0.98	1.05			
Gender	1.18	0.77	1.81			
Comorbid diseases	1.16	0.75	1.79			
Complications	.90	.68	1.19			

^a^ *p* < .10.

^b^ *p* < .05.

Note *R*^2^ = .35 (Nagelkerke), Hosmer and Lemeshow Goodness of Fit *χ*^2^(8) = 3.83, *p* = .87.

**Table 5:** Unadjusted and adjusted logistic regression analyses of variables associated with Depressive symptoms (EK10-depression).

Predictor	Unadjusted OR	95% C.I.		Adjusted OR	95% C.I.	
		Lower	Upper		Lower	Upper
Ethnicity						
Moroccan	*3.54* ^a^	1.71	7.31	1.56	0.47	5.17
Turkish	*5.09* ^a^	2.12	12.19	*4.23* ^a^	1.05	17.04
Surinamese	*2.47* ^a^	1.13	5.42	1.95	0.61	6.26
Other	0.65	0.26	1.65	0.64	0.18	2.21
Socioeconomic status	*2.10* ^a^	1.24	3.55	0.84	0.36	2.00
Age	*0.98* ^a^	0.97	1.00	1.00	0.97	1.03
PCS (SF12)	*0.95* ^a^	0.92	0.97	* 0.96* ^a^	0.93	0.99
MCS (SF12)	0.98	0.95	1.01			
PAID5	*10.43* ^a^	5.91	18.40	* 7.12* ^a^	3.11	16.33
EK10-anxiety	*18.68* ^a^	8.67	40.25	* 10.63* ^a^	3.93	28.78
HbA1c	*1.02* ^a^	1.00	1.04	1.00	0.97	1.03
BMI	1.03	0.99	1.07			
Gender	1.09	0.65	1.84			
Comorbid diseases	1.04	0.62	1.76			
Complications	1.10	0.79	1.53			

^a^ *p* < .05.

Note *R*^2^ = .50 (Nagelkerke), Hosmer and Lemeshow Goodness of Fit *χ*^2^(8) = 4.55, *p* = .80.

**Table 6:** Unadjusted and adjusted logistic regression analyses of variables associated with anxiety symptoms (EK10-anxiety).

Predictor	Unadjusted OR	95% C.I.		Adjusted OR	95% C.I.	
		Lower	Upper		Lower	Upper
Ethnicity						
Moroccan	*3.54* ^b^	2.97	5.05	1.15	0.47	2.81
Turkish	*2.22* ^b^	1.06	4.64	0.67	0.20	2.21
Surinamese	*2.30* ^b^	1.32	3.99	1.51	0.65	3.47
Other	.94	0.59	1.51	0.91	0.48	1.75
Socioeconomic status	*1.98* ^b^	1.40	2.82	*1.98* ^b^	1.15	3.40
Age	.99	0.98	1.01			
PCS (SF12)	*.97* ^b^	0.95	.98	0.99	0.97	1.01
MCS (SF12)	.99	0.97	1.01			
PAID5	*6.55* ^b^	4.05	10.62	*2.63* ^b^	1.33	5.20
EK10-depression	*18.68* ^b^	8.67	40.25	*9.91* ^b^	3.77	26.05
HbA1c	*1.02* ^b^	1.01	1.03	*1.02* ^b^	1.00	1.04
BMI	1.02	1.00	1.05			
Gender	*1.38* ^a^	0.98	1.93	1.35	0.82	2.22
Comorbid diseases	1.23	0.88	1.73			
Complications	*1.36* ^a^	0.95	1.94	1.33	0.77	2.27

^a^ *p* < .10.

^b^ *p* < .05.

Note *R*^2^ = .31 (Nagelkerke), Hosmer and Lemeshow Goodness of Fit *χ*^2^(8) = 5.17, *p* = .74.
